# *Systemic* Wound Healing Associated with *local* sub-Cutaneous Mechanical Stimulation

**DOI:** 10.1038/srep39043

**Published:** 2016-12-23

**Authors:** Christine Nardini, Valentina Devescovi, Yuanhua Liu, Xiaoyuan Zhou, Youtao Lu, Jennifer E. Dent

**Affiliations:** 1 Group of Clinical Genomic Networks, Key Laboratory of Computational Biology, CAS-MPG Partner Institute for Computational Biology, Shanghai Institutes for Biological Sciences, Shanghai 200031, P.R. China; 2 CNR IAC “Mauro Picone”, Via dei Taurini 19 00185-Roma, Italy; 3 Bioinformatics Platform, Institut Pasteur of Shanghai, CAS, Shanghai 200031, P.R. China; 4 NORSAS consultancy limited, Norwich (NR12 8QP), Norfolk, UK

## Abstract

Degeneration is a hallmark of autoimmune diseases, whose incidence grows worldwide. Current therapies attempt to control the immune response to limit degeneration, commonly promoting immunodepression. Differently, mechanical stimulation is known to trigger healing (regeneration) and it has recently been proposed locally for its therapeutic potential on severely injured areas. As the early stages of healing consist of altered intra- and inter-cellular fluxes of soluble molecules, we explored the potential of this early signal to spread, over time, beyond the stimulation district and become systemic, to impact on distributed or otherwise unreachable injured areas. We report in a model of arthritis in rats how stimulations delivered in the subcutaneous dorsal tissue result, over time, in the control and healing of the degeneration of the paws’ joints, concomitantly with the systemic activation of wound healing phenomena in blood and in correlation with a more eubiotic microbiome in the gut intestinal district.

Mechanosensing is a pervasive ability of living cells, however, the informed exploitation of this characteristic is still in its infancy. Mechanical stimulation is a potent trigger to epithelial-mesenchymal transition (EMT ref. 1), a ubiquitous phenomenon^2^ at the base of events as diverse as embryonic development (EMT type 1), wound healing (EMT type2) and tumor progression (EMT type 3). Pioneering work towards therapeutic usage of this property touches applications such as muscle regeneration^3^ and tumor growth^4^, however, a broader and systematic involvement of mechanical stimulation in the control of biological functions is far from routine practice.

Recently, we prospected^5^ to exploit the heterogeneity of the *omics* technologies to explore -spatially and temporally across the patient’s body- the effects of a therapeutic mechanical stimulation as the biochemical signal transduced from the point of stimulation to a distinct disease target organ, across the blood and the gut intestinal (GI) microbiome systemic districts, crucial players and carriers of a wide variety of biochemical signals for immune and metabolic processes. We here present the results of this investigation tested on a model of rheumatoid arthritis (Collagen Induced Arthritis, CIA^6^) induced in animals (Wistar rats).

The study includes untreated healthy animals (NOCIA) and CIA animals cured with: methotrexate (MTX, gold standard^7^), mechanical stimulation (MS, subcutaneous dorsal stimulation), placebo for MTX (PLA, sterile saline solution (0.9% NaCl)) and control inhalational anesthetic (ANE, isoflurane, versus ether used in PLA, MS and MTX during animals’ blood sampling).

## Results

### Phenotypic and PBMC molecular surrogates

**Figure 1:**
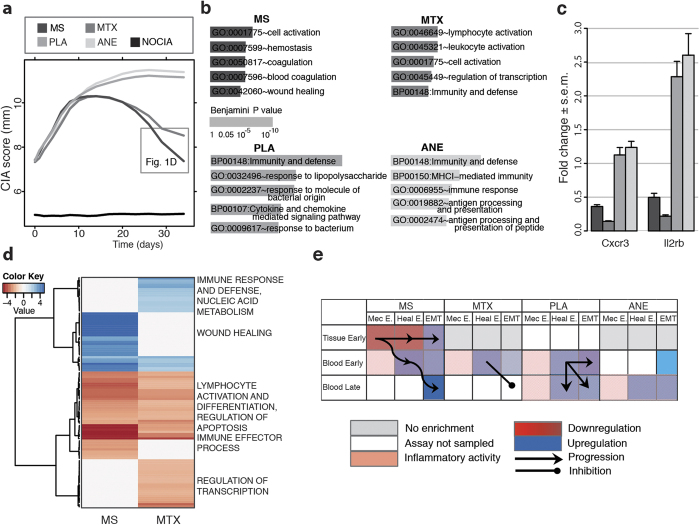
Phenotypic and PBMC characterization. (**a**) Standard clinical parameters (paws’ thickness, CIA score, mm) in the MS, MTX, PLA, ANE and NOCIA arms over time (34 days). (**b**) Significant (top 5) enriched functional categories, for differential genes (*limma* FC > 2, P value < 0.05), therapy-wise. The inflammatory response dominates in control arms (PLA, ANE). (**c**) Validation by qRT-PCR (Supplementary Data S2) of cytokines representative of the differential inflammation ongoing in control (PLA, ANE) versus active therapy arms (MS, MTX). s.e.m. = standard error of mean. (**d**) Heatmap of the differential and functional analysis focused on the comparison MTX-MS. (**e**) Summary enrichment analysis for wound healing (EMT type2). Color intensity is proportional to the number of tested variants (molecular, functional, methodological and experimental) that confirm the enrichment of the spatiotemporal sampling (subcutaneous tissue 1 h, PBMC 1 h and 34 days) for the EMT temporal phases (Mec. E. stands for early mechanotransduction, Heal E. for early healing).

Paws’ qualitative and quantitative thickness collected for standard evaluation of CIA’s onset and progression (CIA scores, Fig. 1a, Supplementary Data S1) shows that active (MS, MTX) and control (ANE, PLA) therapies form statistically significantly distinct groups (Supplementary Table S1). Peripheral blood mononuclear cells (PBMCs) molecular surrogates of these phenotypes were identified therapy-wise, with functional analysis of high-throughput screens. Results highlight increasing (PLA, ANE) and regressing (MTX, MS) inflammatory response (Fig. 1b, Fig. S2 and Supplementary Data S2), confirmed by qRT-PCR (Fig. 1c) and independent experiments (Supplementary Data S1). The statistical analysis also suggests a time-to-therapy effect whose functional PBMC characterization was explored for the two active treatments (MS versus MTX at day 34, Fig. 1d, Supplementary Table S1 and Supplementary Data S2) highlighting 3 major functional areas: MTX’s distinctive *nucleic acid metabolism* and *regulation of transcription*, in line with known effects of the drug^8^; *lymphocyte activation and differentiation* in both treatments, also in line with MTX immunomodulatory effects^9^; and *wound healing* related processes, peculiar to MS. Wound healing (EMT, type2 ref. 10) is a phenomenon considered to be local to the close surrounding of an injured area^11^, involving bidirectional signaling (including at later stages lymphocytes activation) from tissue cells to proximal hematocytes, and developing over time into inflammation, regeneration and remodeling^1^.

### Molecular flow

To gain insight into the observed systemic (PBMC, versus local) *wound healing* process, we deepened (Fig. 1d) the functional analysis along: the spatial dimension, adding the sampling of the dorsal subcutaneous tissue (Supplementary Data S3); the molecular diversity, with post-transcriptional data (miRNAs, Supplementary Data S4–5); the functional complexity, by testing our results against four EMT molecular description variants (Supplementary Data S6); the methodological bias, using 3 types of functional enrichment analysis (GO analysis by DAVID^12^, hypergeometric distribution^13^, GSEA^14^, Supplementary Data S6); the reproducibility of the phenomenon, with a second independent experiment (batch2). We also explored the temporal dimension with the identification of early genes (differential1h after therapy, Supplementary Data S2–5) and their enrichment for *early wound healing* processes, i.e. genes sets activated by non-transcriptional cues (purinergic signaling, reactive oxygen and nitrogen species gradients and Ca^2+^ waves), largely shared by injury (early non-transcriptional phase of wound healing: *Heal.E*^11^) and mechanical stimulation (immediate response to mechanical injury *Mec.E*.^15^).

Figure 1e (Supplementary Data S6) summarizes across these multiple tests - performed to guarantee the robustness of the results- the statistically significant functional activations (EMT phases, columns) that cover a spatiotemporal flow (from subcutaneous tissue to blood, from 1 h to 34 days, rows). Each of the study arms present a distinctive pattern related to a response to injury, embodied in MTX and PLA by the injection (drug for the former, placebo for the latter) and in MS by the insertion and rotation of a thin needle. Previous reports^16^ indicate that rotation is required for a needle mechanical stimulation to be transduced into the Rho/Rac signaling cascade, crucial player in EMT for the cytoskeleton and extracellular matrix reorganization^10^. Coherently we observe in MTX and PLA that the injection activates only the initial response to injury (inflammation), due to the lack of rotation of the syringe needle. In MTX, the absence of this mechanical cue, adds to the known interference of the drug with damage-associated molecular patterns (DAMPs ref. 17) crucial to the transmission of the early healing signal^11^, with both factors contributing to the inhibition of the EMT progression in the late sample (34 days). In PLA the evolution of the disease (CIA) maintains inflammatory processes active, with no other intervention to evolve towards later stages proper of healing. Coherently to this picture, ANE, aerially delivered, and hence lacking of any triggering injury, shows no sign of response to wound. In conclusion, MS alone evolves across all EMT type2 phases, including, importantly, control on the inflammatory activity (molecular details in Supplementary Data S2–5).

### Gut-intestinal microbiome

**Figure 2:**
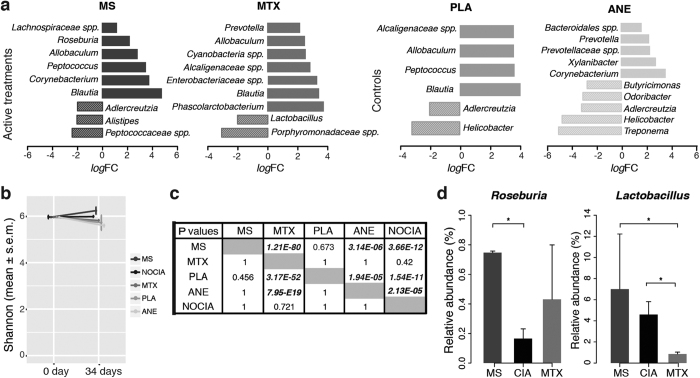
GI microbiome analysis. (**a**) Differential analysis (*limma* FC > 2, P value < 0.05), therapy wise. (**b**) Diversity analysis by Shannon index. (**c**) Contingency test comparing therapy-wise cumulated frequencies (increasing harmful and decreasing unharmful, i.e. dysbiotic variations and decreasing harmful and increasing unharmful, i.e. eubiotic variations) testing the null hypothesis (fisher one-sided) that treatment A (row) leads to a more eubiotic GI composition than treatment B (column). Significant associations (P < 0.05) in bold. (**d**) Genera highlighted for their relevance in the control of inflammation, statistically significant differences are annotated with * (P < 0.05).

To explore further the cues contributing to the overall therapeutic outcome we analyzed the variations of the GI microbiome as a crucial player of the immune response^18^ (differential analysis, Fig. 2a, Supplementary Data S7). The α diversity measured by Shannon index reveals an increase of microbial diversity in CIA samples treated with MS while MTX, PLA and ANE lead to a reduction of microbe diversity (Fig. 2b). The low diversity of microbes within the GI district has already been related with obesity and inflammatory bowel disease^19^,^20^ and in general it is an index of the paucity of functions that can be activated by the GI microbiome^21^. To gain further insight into the therapeutic impact of such diversity, we adopted an unsupervised approach to define the variations in terms of more abundant eubiotic or dysbiotic genera, with the collection of the relative decrease of harmful bacteria and/or increase of unharmful ones (*eubiotic* frequencies) versus the opposite (*dysbiotic* frequencies). Frequencies organized therapy-wise in contingency tables were used to test (Fisher and *χ*^*2*^) whether the *variation* of the composition of the GI microbiome associated to a therapy is more likely to lead to a eubiotic microbiome than an alternative treatment (Fig. 2c and Supplementary Data S7). Unsurprisingly, healthy animals (NOCIA) do not present meaningful variations in any direction (eubiotic/dysbiotic) over the course of the experiment (34 days). PLA is associated to variations that are more eubiotic than MTX, ANE and NOCIA, a finding relevant to the current research on the impact of anaesthetic (isoflurane for ANE and ether for PLA) on oxidative stress and inflammation^22^ known cues able to shape the GI microbiome composition^23^. MTX shows no positive association to a eubiotic microbiome, suggesting that the action of MTX, capable to contrast more strongly the joints degeneracy by apoptosis, shows a flip side at the GI microbiome level, additional, or synergistic, with its immunosuppressive effects, acknowledged as the MTX-induced enterocolitis^24^. In fact, although in our experiments both MTX and MS show a general reduction of inflammation in the bloodstream, MTX is known to induce oxidative stress in the small intestine^25^, known cause of dysbiosis^26^. Conversely, variations computed in the MS arm show significant positive association to a more eubiotic microbiome in comparison with MTX, ANE and NOCIA. At the genus level (Fig. 2d, Supplementary Data S7), *Lactobacillus*, a known probiotic capable to alleviate platelets activation in inflammatory pathways^27^ is observed to be markedly decreased in MTX (and differential between MS and MTX), while *Roseburia* increased in MS only, is a producer of butyrate^28^ known to control inflammation and oxidative stress by inhibiting a number of pro-inflammatory pathways including NF-kB^29^.

### Target organ: joints synovium

To complete the ideal signaling flow from the stimulation point (dorsal subcutaneous tissue) to the systemic districts (PBMC, GI microbiome) and finally to the disease target organ (joints) we tested in a simplified setup the events occurring in the paws’ synovium, by co-culture of RA fibroblasts like cells (RASFLs) at CIA onset with: functional blood (harvested halfway of the MS treatment, MS+, day 18), non-functional blood (MS−, harvested in untreated, CIA induced animals), and fetal bovine serum (FBS) for control.

**Figure 3:**
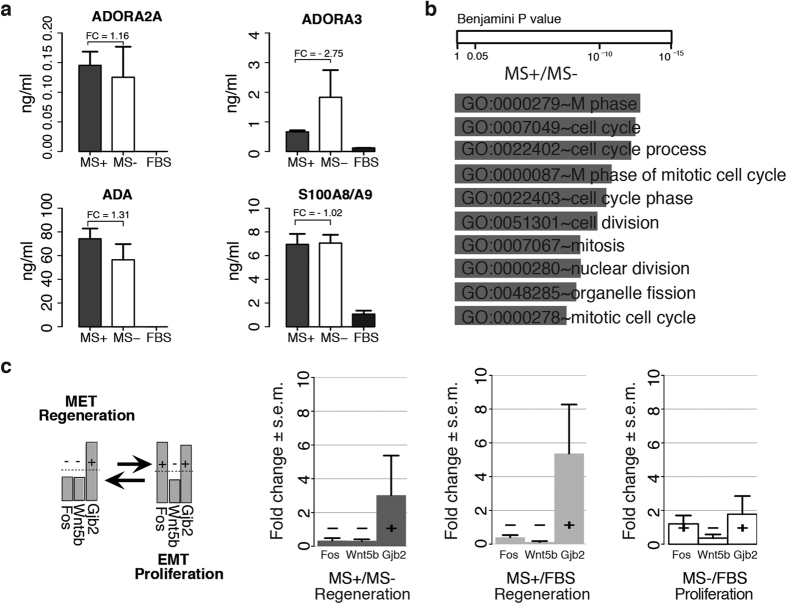
Joints Rheumatoid Arthritis synovium fibroblast-like (RASFL) analysis. (**a**) ELISA assay on ADORA2A, ADORA3, ADA, S100A8/A9 levels in the blood serum of MS treated (MS+), untreated (MS−) animals and in control (FBS). (**b**) Functional analysis of differentially expressed genes in MS+ versus MS− (FC > 2). (**c**) Left panel: EMT/MET conformation of epithelial/mesenchymal markers; right panel: expression fold changes (mean ± s.e.m.) of *Fos, Wnt5b* and *Gjb2*. The +/− located at FC = 1, highlights the regulation direction.

Functionality of the blood was tested on ADORA2A, ADORA3, ADA and S100A8/A9 given the known importance of purinergic signaling in (model) arthritis^30^. Figure 3a show that ADORA3, a systemic and target organ biomarker for (model) arthritis –with ADORA3 agonists under test for RA therapy^31^ – is distinctively reduced (>2 fold change) in functional blood (MS+) compared with non-functional blood (MS−). The transcriptomic effects (Fig. 3b and Supplementary Data S8) of the 3 serological settings (MS+, MS−, FBS) refer to c*ell cycle* activation with emphasis on the mitotic phase, indicating positive survival signals received by the cells towards an active division and duplication. This translates equally likely in RASFLs as a message of *proliferation,* proxy for *tissue invasion* (synovial hyperplasia, cell over-proliferation in an inflammatory milieu), or *tissue regeneration* (homeostasis and repair, cell proliferation in a physiologic microenvironment), corresponding to a transition towards a mesenchymal or epidermal phenotype, respectively^32^. To disambiguate the epithelial versus mesenchymal conformation, three distinctive key molecules, *Fos, Wnt5b* and *Gjb2*, were validated by qRT-PCR. The epithelial conformation (Fig. 3c) implies control (downregulation) of *Fos,* whose activation causes epithelial cells to gain the mesenchymal characteristics of high-proliferative rate, loss of polarity and invasiveness via the *Smad* signaling^33^; reduced expression of *Wnt5b,* whose activation triggers the mesenchymal Wnt pathway^34^; and upregulation of *Gjb2,* a cell membrane protein crucial for maintaining cellular adhesion, growth control and tissue homeostasis, typically expressed by epithelial-like cells^35^. Figure 3c shows that MS+ is characterized by a general repression of the mesenchymal phenotype in favor of the epithelial one. This is in line with the observed reduction of ADORA3 and inflammation in several chronic inflammatory diseases and cancer^36^ and the exploitation of ADORA3 agonists to control cell cycle in therapeutic contexts where over-proliferation is involved^37^.

The flow of information that we here describe permits to read the control on CIA symptoms as the different effects of the multifaceted phenomenon of epithelial mesenchymal transition type2, investing not only the district associated to the mild therapeutic injury (needle insertion and rotation), but spreading to the whole organism, via small signaling molecules, typical of early healing and mechanotransduction, conditioning the biochemical signaling in blood in continuous crosstalk with the GI microbiome, and hence globally defusing the inflammatory environment typical of the disease. This promotes a virtuous control on the immune response, body-wise, and as a consequence in the special district target of the disease: the joints synovium, leading to a traceable reduction of a specific systemic arthritis biomarker like ADORA3.

Overall, the effects of a mechanical stimulation observed in this systemic molecular perspective return a chain of plausible cause-effect molecular events referable to the known categories of mechanotransduction and wound healing, complementing previous knowledge on other manipulative therapies^38^.

This approach presents limited invasiveness, and in the exemplar case of RA it tackles the problem of degeneration from a perspective complementary to mainstream treatments. Disease modifying anti-rheumatic drugs (DMARDs) therapy of election in (model) RA, in fact, control degeneration indirectly by disrupted (immune) pathways’ blockade (targeted for biological DMARDs and untargeted for conventional DMARDs like MTX). MS complementarily, promotes a return to homeostasis by activating self-healing processes. The former approach is usually stronger in term of phenotypic changes than the latter, but also more partial (being effective somewhere downstream of the origin of the disease) and hence more prone to negative and uncontrollable side effects. Translation in humans and further characterization of the phenomenon may find broad applicability in evidence based medicine for the treatment of autoimmune and degenerative diseases.

## Methods

### Study design

The studies were approved by Animal Ethics Committee of Zhongshan Hospital, Fudan University (Shanghai, China), performed with methods in accordance with the relevant guidelines and regulations and all measures were taken to minimize animal number and suffering. For batch1: Fifty-five Specific Pathogen Free (SPF) female Wistar rats were purchased from the Animal House Centre of Fudan University (Shanghai, China). Rats were housed 5 per cage, maintained in an environmentally controlled room with 12 hour light/dark cycle and kept on a standard chow and water *ad libitum*. A second experiment (batch 2) was run for validation months apart with twenty-three SPF female Wistar rats purchased, raised and induced in the same conditions as follows.

Animals were either anaesthetized and immunized with 0.1 mL intradermal injection at the base of the tail, or 2 mg/ml Bovine type II collagen (Chondrex) dissolved in 0.05 M acetic acid and emulsified 1:1 Complete Freund’s Adjuvant (Sigma-Aldrich, DK). A booster injection of Bovine type II collagen prepared as previously described and emulsified 1:1 Incomplete Freund’s Adjuvant (Sigma-Aldrich, DK), was given on day 7 after initial immunization (Protocol for the Successful Induction of collagen induced arthritis (CIA) in Rats, Chondrex, Inc. 2009). Arthritis (CIA) score from 0–4 was attributed depending on the swelling according to standard practice^6^. A thickness gauge (Myouto, JP) was placed on the tarsal (dorsal/ventral) of the hind paw and thickness was determined a minimum of two times to ensure accuracy, with the same frequency of the quantitative visual inspection. Hundred percent of rats developed arthritis; onset of the disease was declared at day 18 after immunization for CIA score ≥2 and treatment initiated. Every other day, starting from day 8 (18 time points in total), rats were examined for visual signs of disease, defined as macroscopic evidence of increase in hind paw size, determined paw score. See Supplementary Data S1.

The MS group received mechanical stimulation induced with a ring-headed thumb-tack like stainless-steel needle (ϕ0.25 mm*2 mm, diameter*length, Hwato, Suzhou, P.R.C.) by punction and 20 s clockwise twirling at the beginning of each treatment session (20 m) (Supplementary Fig. S1). Punction was applied bilaterally on points located at each side of the lower back spine between the 2^nd^ and the 3^rd^ lumbar vertebra, and between the tibia and fibula at approximately 5 mm lateral and 5 mm lower to the anterior tubercle of the tibia^39^ (these points are also known in traditional Chinese medicine as “Shenshu” (BL 23) and “Zusanli” (ST 36), respectively, chosen based on medical doctors’ expertise, see Acknowledgments, with no *a priori* in our experimental design on the relevance of the localization). In batch1, the MTX group received a peritoneal injection of the methotrexate (0.3 mg/kg) once a week; the PLA group received a sterile saline (0.9% NaCl) peritoneal injection with the same frequency. Animals used as anesthesia control (ANE) were anesthetized with a different drug (Isoflurane, versus ether) during blood sampling and received no other therapy. In batch2, the MTXMS group received both therapies (MTX and MS) with the same modalities of the separate therapies; NOCIAMS received MS with the same modalities as MS on healthy animals (no CIA). As MS animals needed shaving at stimulation points for therapy delivery, all animals were shaved to minimize sources of bias. In batch2 the MS therapy was replicated as for batch1, MTXMS consists of the administration of both MTX and MS treatments, NOCIAMS implies MS as described already and delivered to healthy animals. Details on the overall design of experiments can be found in Supplementary Table S2.

### Fecal, blood, subcutaneous tissue samples collection

Fecal specimens were collected before blood sampling in sterile vials and rapidly frozen at −20 °C and further stored at −80 °C (batch 1). Whole blood sampling for high-throughput screens (microarray) was carried out before any therapy for the CIA and NOCIA groups (CIA_B and NOCIA_B). All groups (MS, MTX, PLA, ANE and NOCIA) were additionally sampled at 1 hour and 34 days (respectively: Early and After therapy: MS_E, MS_A, MTX_E, MTX_A etc.). Animals were induced in deep general anesthesia and after total blood drawing (5 ml), sacrificed by neck dislocation. In batch2, the early time point (1 h) was excluded. Subcutaneous tissue surrounding the stimulated points were excised upon animal sacrifice (time zero, 1 hour, 34 days), with surgical scalpel and specimens were rapidly nitrogen liquid treated before storing at −80 °C.

### Fecal samples microbial shotgun sequencing

Microbial DNA was extracted from fecal samples using the E.Z.N.A. Soil DNA Kit (Omega Bio-tek, Norcross, GA, U.S.) according to manufacturer’s protocols. The V1-V3 region of the bacteria 16 S ribosomal RNA gene were amplified by polymerase chain reaction (95 °C for 2 min, followed by 25 cycles at 95 °C for 30 s, 55 °C for 30 s, and 72 °C for 30 s and a final extension at 72 °C for 5 min) using primers 27F 5′-(CGTATCGCCTCCCTCGCGCCATCAG-3′ 5′-AGAGTTTGATCCTGGCTCAG)-3′ and 533R 5′- (CTATGCGCCTTGCCAGCCCGCTCAG- 3′ -MID tags-5′-ATTACCGCGGCTGCTGGCA)-3′. PCR reactions were performed in a 20 μL mixture containing 4 μL of 5 × FastPfu Buffer, 2 μL of 2.5 mM dNTPs, 0.8 μL of each primer (5 μM), 0.4 μL of FastPfu Polymerase, and 10 ng of template DNA.

After purification using the AxyPrep DNA Gel Extraction Kit (Axygen Biosciences, Union City, CA, U.S.) and quantification using QuantiFluor™ -ST (Promega, U.S.), a mixture of amplicons was used for pyrosequencing on a Roche 454 GS FLX (Roche 454 GS FLX + for batch2) Titanium platform (Roche 454 Life Sciences, Branford, CT, U.S.) at a depth of 10000 reads per sample in batch1 (and 15000 reads per sample in batch2) according to standard protocols at Majorbio Bio-Pharm Technology Co., Ltd., Shanghai, China.

### Total RNA and protein extraction from blood

Peripheral blood mononuclear cells were freshly isolated on Ficoll gradient (Histopaque-1077, Sigma Aldrich, USA) according to the manufacture procedures. Separated cells were maintained in RNA Later (Ambion, USA) at −80 °C until the end of the study and then processed in a whole batch for the following extraction. Total RNA and proteins were purified from each cell sample with MirVana PARIS kit (Ambion, USA) and the concentration measured by Nanodrop 2100 (Thermo Scientific, DK).

### Total RNA extraction from subcutaneous tissue samples

Subcutaneous tissue samples (including subcutaneous muscle, <500 mg) were excided under deep general anesthesia, and immediately dipped in liquid nitrogen for 1 minute then moved and maintained at −80 °C until use. Total RNA was extracted and purified from dry frozen tissue quickly dipped in Trizol (Cat#15596-026, Invitrogen, USA) homogenized with a 5 mm blade tip (IKA, DE) and processed according total RNA extraction protocol. The RNA obtained was quantified by Nanodrop 2100 and tested for standard quality parameters (RIN, RNA Integrity Number) through Total RNA 6000 Nano kit (Cat#5067-1511, Agilent, DE). The RIN qualified RNA samples were sent to Illumina Sequencing Services of Partner Institute for Computational Biology Omics Core and cDNA libraries were constructed for sequencing as described in Illumina TruSeq™ RNA sample preparation v2 guide (Catalog # RS-930–1021). Sequencing was performed by Illumina HiSeq 2000.

### Real-time quantitative PCR

To validate microarray results on PBMC (batch1) qRT-PCR was performed in house. Total RNA was used to synthesize cDNAs using a reverse transcription kit according to the manufacturer’s instructions (Takara, JP). The obtained cDNA was then used as a template for analyzing the selected genes using real-time quantitative PCR (Roche Diagnostic, USA). GAPDH was used as calibrator for total RNA input (250 ng) and to quantify gene of interest expression. On batch2 qRT-PCR to validate microarray results and the 3 proliferative/regenerative markers in RASFLs was outsourced. Reverse transcriptase reactions from RNA to cDNA were carried out with Taqman MicroRNA Reverse Transcription Kit (ABI, USA). Real-time quantitative PCR was then performed using Taqman Universal PCR Master Mix (ABI, USA). GAPDH was used as a control for total RNA input (0.75 μL per 10 μL reaction) and to quantify gene of interest expression. Fold-change (FC) and error range calculation was performed as the ratio of the averaged gene expression (on technical replicates and relative to the calibrator gene Gapdh) between two groups: 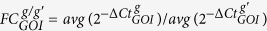.

The error range is the standard error of the mean of the relative gene expression in both groups:


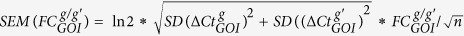


where *g, g*′ represent 2 treatment groups, *GOI* the gene of interest, *n* the number of biological replicates^40^. Data stored in Supplementary Data S2, S8.

### *In vitro* culture

RA synovial fibroblasts like cells (RASFLs) were obtained from synovium of CIA induced rats at time zero (CIA score ≥2). Fresh synovial tissue was minced and digested for 5 h with 0,2% of type I collagenase (Sigma Aldrich) in serum-free Dulbecco’s modified Eagle’s medium, DMEM (Invitrogen) at 37 °C with 5% CO_2_ content, on a horizontal shaker. The suspension was then filtered, washed twice with PBS (Gibco) and plated in 75 cm^2^ tissue culture flasks. After overnight culture, non-adherent cells were removed, and adherent cells were maintained in complete medium, i.e. DMEM supplemented with 10% fetal bovine serum (FBS), 2 mM L-glutamine, 100 U/ml of penicillin, 100 μg/ml streptomycin, HEPES (Sigma Aldrich). The cultures were kept at 37 °C, in 5% CO_2_ atmosphere and fresh medium was replaced every 3 days. RAFLs cultures were expanded and used for experiment at passage 5^41^.

Cells were divided in three groups and maintained in different culture conditions, i.e. medium added with: (i) FBS; (ii) rats’ blood serum from MS treated animals (MS+); rats’ blood serum from untreated animals (MS−). Blood serum was previously withdrawn from MS treated animals at day 18 (MS+), and before any therapy was initiated (MS−). The samples were collected in sterile tubes, spun at 1000× g for 10 min and stored at −80 °C until use.

After 72 h cells were harvested enzymatically with trypsin (Pierce), collected by centrifuge and re-suspended in RNA Later (Invitrogen) before −80 °C storage^42^. RNA-seq was done by BGI-Shenzhen Company with total RNA samples first treated with Dnase I to degrade any possible DNA contamination. Further mRNA was enriched by using oligo(dT) magnetic beads, mixed with fragmentation buffer, and fragmented into short segments (~200 bp). The first strand of cDNA was synthesized with random hexamer-primer. Buffer, dNTPs, RNase H and DNA polymeraseI were added to synthesize the second strand. The double strand cDNA was purified with magnetic beads, reparation and 3′-end single nucleotide A addition was performed. Sequencing adaptors were ligated to the fragments, finally enriched by PCR amplification. Agilent 2100 Bioanalyzer and ABI StepOnePlus Real-Time PCR system were used to qualify and quantify the sample library to be used with Illumina HiSeqTM 2000.

### Enzyme-linked Immunosorbent Assay (ELISA)

Four proteins were quantified by ELISA: Adenosine A2A receptor (ADORA2A, antibodies-online, Aachen, Germany), Adenosine A3 Receptor (ADORA3, Cloud-clone Corp., Houston, USA), Adenosine Deaminase (ADA, BlueGene Biotech CO., Shanghai, China) and S100 Calcium Binding Protein A8/A9 (S100A8/A9, BlueGene Biotech CO., Shanghai, China) in 3 different sera: MS+ (3 biological replicates of blood serum from MS treated animals); MS− (3 biological replicates of blood serum from untreated animals); FBS (3 biological replicates as control, in fetal bovine serum). All standards and samples were assayed in duplicate, following the manufacturer’s indication and read spectrophotometrically (optical density, OD) with Bio-Tek synergy H1 microplate reader. Curves for standards ODs/concentrations assay were fit with a 4 parameters logistics (4-PL) model. Fold changes were calculated for comparisons in the 3 different experimental conditions (MS+, MS−, FBS) as the ratio of protein concentration averaged among three biological replicates with a cutoff of 2-fold^43^.

### Omics differential analysis

After pre-processing specific to each *omic* (see Supplementary Methods) differential analysis was performed using limma^44^ for microarray data and the pipeline limma + voom^45^ RNA/16SrRNA-seq data, using for threshold a combination of foldchange (FC > 2) and P value (P < 0.05).

### PBMC miRNA and mRNA functional analysis

Differential mRNAs were annotated using DAVID, with corrected P value (Benjamini) <0.05, and background *Rattus norvegicus*. The miRNAs were annotated based on their targets identified as predictions via miRDB^46^ with score 80 as described in ref. 47 and identified as experimentally validated with miRBase v18^48^. Functional analysis was also done on the union of differential miRNA targets (predicted and validated) and differential mRNAs as in ref. 47.

### EMT gene sets

Four sets were manually compiled to capture temporal and functional phases of EMT: (i) Heal. E. for early healing, based on^11^ with unspecific downstream effectors (SRC, PI3K, JNK, ERK) removed; (ii) Mec. E. for early mechanotransduction, from the *core* map in ref. 15 including the early (~300 ms^49^) signaling from the force to the activation of SRC and F-Actin; (iii) EMT from SAB website (http://www.sabiosciences.com/Biomarker.php, PARN-090Z) (iv) EMT.T2 from (iii), with EMT Type1 and Type3 specific markers removed, Type2 specific markers added^2^,^50^. EMT type2 associated GO terms *Wound Healing* (GO: 0042060, whGO) and *Response to wounding* (GO: 0009611, rwGO) where expanded in the form of genes lists, the same was done for *Inflammation* (GO: 0006954, InflGO). All sets are collected in Supplementary Data S6 sheet “Functions (Genesets)”.

### Phenotypic data ANOVA

The 18 observations of the qualitative (categorical) arthritis score were transformed to a scale of 1–5. The quantitative data were preserved on their continuous scale. For both datasets, the NOCIA data were not considered and missing data were omitted. Only rats that received therapy were included in the analyses. Generalized linear models were fitted to the data to describe arthritis scores measurement over time for different treatments. To test for a treatment group effect, data were centered (around the mean) and pooled on a pairwise basis, GLMs were fitted (assuming a maximum of t^4^) and ANOVA was used to test for a significant group effect. The same analysis was performed on Batch2 (Supplementary Data S1 and S2).

Analyses are presented for the continuous data only, and are confirmed on discrete data (Supplementary Data S1 contains categorical data in addition to continuous data).

### Association study between therapy and microbes

OTU-based alpha diversity was evaluated by Shannon index and averaged among samples in each arm for comparisons between CIA and treated CIA arms. Differential genera were labeled as *unharmful, harmful* based on literature or remained *unknown,* when defined so in the differential analysis. A genus was annotated as harmful when harmful species were included in that genus and unharmful when no harmful species were included, based on the observation that although genera may vary, species are robust with respect to these definitions (harmful or unharmful). The TMM normalized genus’ abundance, computed in the differential analysis step (before log2-cpm conversion, needed for *limma* linear model differential analysis, Supplementary Methods), was used to compute a genus’ *increase* or *decrease* as the difference of the after versus before therapy abundance. Unharmful genera that appear to be more abundant and harmful genera that result as less abundant after therapy, jointly (summed absolute values, eubiotic frequency) define an improvement towards a eubiotic (beneficial) microbiome. The reverse holds (less abundant unharmful genera and more abundant harmful genera, summed absolute values, dysbiotic frequency) for the definition of a more dysbiotic microbiome. Unknown genera are removed from this analysis. Contingency tables, using chi-square and Fisher’s exact test^13^, were employed to assess the null hypothesis that the proportions of eubiotic microbe frequencies are different between therapies. One sided Fisher’s exact test was then performed to assess the positive association of a therapy with a more eubiotic GI microbiome^51^.

### Data and materials availability

O*mic* data accessible at GEO (http://www.ncbi.nlm.nih.gov-/geo/) and SRA (http://www.ncbi.nlm.nih.gov/Traces/sra/) with specific IDs and all other data in Supplementary Information.

## Additional Information

**How to cite this article**: Nardini, C. *et al*. *Systemic* Wound Healing Associated with *local* sub-Cutaneous Mechanical Stimulation. *Sci. Rep.* **6**, 39043; doi: 10.1038/srep39043 (2016).

**Publisher's note:** Springer Nature remains neutral with regard to jurisdictional claims in published maps and institutional affiliations.

## Supplementary Material

Supplementary Information

Supplementary Dataset 1

Supplementary Dataset 2

Supplementary Dataset 3

Supplementary Dataset 4

Supplementary Dataset 5

Supplementary Dataset 6

Supplementary Dataset 7

Supplementary Dataset 8
