# Corrigendum: *Systemic* Wound Healing Associated with *local* sub-Cutaneous Mechanical Stimulation

**DOI:** 10.1038/srep46118

**Published:** 2017-04-05

**Authors:** Christine Nardini, Valentina Devescovi, Yuanhua Liu, Xiaoyuan Zhou, Youtao Lu, Jennifer E. Dent

Scientific Reports 6: Article number: 3904310.1038/srep39043; published online: 12 23 2016; updated: 04 05 2017

Affiliation 1 was incorrectly listed as ‘Group of Clinical Genomic Networks, Key Laboratory of Computational Biology, CAS-MPG Partner Institute for Computational Biology, Shanghai Institutes for Biological Sciences, Shanghai 200031, P.R. China’ in the original version of the Article. The correct affiliation is listed below:

CAS-MPG Partner Institute for Computational Biology, Shanghai Institutes for Biological Sciences, Shanghai, 200031, P.R. China.

In addition, an additional affiliation for all the authors was omitted. The correct affiliation is listed below:

University of Chinese Academy of Sciences, Beijing, 100049, P.R. China.

As a result, the affiliation list and affiliated authors now read:

Affiliation 1

CAS-MPG Partner Institute for Computational Biology, Shanghai Institutes for Biological Sciences, Shanghai, 200031, P.R. China.

Christine Nardini, Valentina Devescovi, Yuanhua Liu, Xiaoyuan Zhou, Youtao Lu & Jennifer E. Dent

Affiliation 2

University of Chinese Academy of Sciences, Beijing, 100049, P.R. China.

Christine Nardini, Valentina Devescovi, Yuanhua Liu, Xiaoyuan Zhou, Youtao Lu & Jennifer E. Dent

Affiliation 3

CNR IAC “Mauro Picone”, Via dei Taurini 19 00185-Roma, Italy.

Christine Nardini

Affiliation 4

Bioinformatics Platform, Institut Pasteur of Shanghai, CAS, Shanghai 200031, P.R. China.

Yuanhua Liu

Affiliation 5

NORSAS consultancy limited, Norwich (NR12 8QP), Norfolk, UK.

Jennifer E. Dent

This has now been corrected in the HTML and PDF versions of this Article.

