# Supplementary material for: Systemic Wound Healing Associated with local sub-Cutaneous Mechanical Stimulation
Source: Sci Rep. 2016 Dec 23;6:39043. doi: 10.1038/srep39043 (PMC5180236; doi:10.1038/srep39043)
Supplement: Supplementary Information [file srep39043-s1.pdf]

Supplementary Information

Systemic Wound Healing Associated with *local* sub-Cutaneous Mechanical Stimulation

Christine Nardini[1,2,*,+], Valentina Devescovi[1,+], Yuanhua Liu[1,3,+], Xiaoyuan Zhou[1,+], Youtao Lu[1,+], Jennifer E. Dent[1,4,+]

Supplementary Methods

Fecal samples differential analysis. Roche 454 pyrophosphate sequencing of the 16S rRNA genes was performed at Majorbio along with: sequences quality control; sequences selection based on samples' barcode identifiers and sequences alignment (SILVA database, SSU106, [1]) to trim chimeras, define Operational Taxonomy Units (OTUs, sequences with 97% similarity) and assign OTUs' taxonomy [2]; calculation of alpha-diversity Shannon index.

OTUs lacking of taxonomic species information were collectively annotated to their closest higher taxonomic level. To limit the impact of the large amount of unknown species OTUs with the same genus classification were merged and genera were considered for further differential analysis. Unannotated genera were labeled *unknown*.

Differential analysis was performed using *limma* [3] where count-based microbial abundance was assimilated to gene expression data [4]. To minimize the noise deriving from low abundance reads and to control for *limma*'s bias in favor of variables with small within groups variance, 2 filtering steps were applied across all the samples of the comparison: genera with null abundance in more than 1 sample and genera with summed abundance below 5, were filtered out [5]. Filtered data were scale normalized with TMM [6] from the *edgeR* package [7] to make samples comparable despite different sequencing depth [8]. Abundance in read counts were converted to log2-cpm

(counts per million) by *Voom* to make them suitable to linear regression in *limma* [9]. Processed and Raw data are available in Supplementary Data S7 and at the National Center for Biotechnology Information Sequence Read Archive database (Accession Number: SRP034624).

PBMC mRNA microarray differential analysis. Affymetrix rat230 2.0 arrays for the 2 batches of experiments were hybridized and scanned according to the manufacturer's recommendations. Raw intensities from .CEL files were analyzed using mas5 from *affy* R package to generate expression intensities for each probe set [10], detection call was calculated using *mas5calls* in the same *affy* package. Before differential analysis, probe-sets were removed if less than 10% of all the samples (independently of the time point and therapy) were detected (absent for detection calls). Expression intensities were transformed in log-2 scale. For all comparisons, significantly differentially expressed genes (DEs) were identified using *limma* [3] with P value <0.05 and foldchange >2. DEs were further removed: i) in case > 2 replicates were available per therapy and time point (batch 2), if 30% of expression intensity (in all samples compared) was not reliable (absent for detection calls); ii) in case ≤ 2 replicates were available (batch1) any of the expression intensity was not reliable.
Data is available at the National Center for Biotechnology Information Gene Expression Omnibus (batch1 GSE48025 and batch2 GSE58456).

PBMC miRNA microarray differential analysis. Agilent Rat miRNA (8*15K) V16.0 arrays were hybridized and scanned according to the manufacturer's recommendations. Data were extracted with Feature Extraction 10.7. Raw signals were then summarized with package *AgiMicroRNA* as previously described [11]. A miRNAs was assumed to be present when any of the

20-40 probes was detected. For each comparison, differentially expressed miRNAs were selected using the same methodologies used for mRNA profiling. Data is available at the National Center for Biotechnology Information Gene Expression Omnibus (GSE58458).

Subcutaneous and Synovium Tissue (RAFLs cells) mRNA Differential Analysis

Quality assessment was confirmed by *FastQC* [12] and *fastx* toolkits [13], including per-base quality score, nucleotide composition, N- and GC content, overrepresented sequences (frequency > 0.1%). Unique read frequency saturation curve was also calculated. Reads filtering was performed differently for subcutaneous tissue and synovium. For subcutaneous tissue data raw reads were trimmed and filtered by *cutadapt* [14]: reads beginning with the 5' adapter (P5) were discarded as PCR artifacts (Hiseq initiates reading after P5) and the trailing 3' adapters (P7) were trimmed (maximum mismatch rate 0.1, overlap length 5), low-quality ends (Phred quality score < 30) were trimmed and remaining reads shorter than 25nt were removed.

For synovium filtered reads include: 1) reads with adapter sequences, 2) reads in which the percentage of unknown bases (N) is greater than 10%, 3) reads in which more than 50% bases quality value ≤ 5, as processed by BGI Shenzhen Company.

Filtered reads were aligned to the rat reference genome (UCSC rn4) using *bowtie2* [15] for subcutaneous tissue and synovium. Gene expressions were quantified by HTSeq v0.5.4p3 [16] at the transcript level using "intersection-nonempty" mode and the UCSC rn4 annotation (downloaded from iGENOME for *rattus norvegicus*). Uniformly to the 16S rRNA-seq processing, raw counts were normalized to cpm by TMM normalization [6] using *edgeR* v3.4. 2 [7] and differential analyses were run with the *limma-voom* pipeline v3.18.9 [3]. Prefiltering for *limma* was done by removal of transcripts with low (more than 20% of replicates < 1 cpm in both groups) or invariant expressions. DEs were selected with: 1) average expression in cpm ≥1; 2)

nominal P value < 0.05; 3) FC ≥2 (output by *limma* as “logFC”). Data is available at the National Center for Biotechnology Information Gene Expression Omnibus (subcutaneous tissue batch1 GSE57983, synovium GSE58978).

Subcutaneous miRNA-Seq differential analysis. Total RNA was used to generate small RNA libraries with Illumina TruSeq Small Sample Prep Kit. Deep sequencing was performed with Illumina Hiseq 2000 at CAS-MPG Partner Institute for Computational Biology Omics Core. Raw sequences were obtained and de-multiplexed using the Illumina pipeline *CASAVA* v1.8. Quality check were performed with *FastQC* [12] and *FASTX* [13] toolkits. The 3’ adapters were trimmed by *mapper.pl* from *miRDeep2* toolkit [17], reads shorter than 18bp were discarded. Clipped reads were aligned using mapper.pl to Rat genome (rn4) and miRBase v18 (rno sequences)[18] and quantified using *quantifier.pl* in miRDeep2. Expression of miRNA data (counts) from all samples were joined and miRNAs with at least 1 cpm in at least half of the total samples were kept. Based on the counts data, the *limma-voom* pipeline [9] was adopted to perform differential analysis, with the same parameters used for mRNA Affymetrix chip data and miRNA Agilent chip data. Data is available at the National Center for Biotechnology Information Gene Expression Omnibus (GSE58459).

Enrichment analysis. To assess the robustness of the results, enrichment tests (*t*) for the differentially expressed genes in the 3 spatiotemporal samples (subcutaneous tissue 1h, PBMC 1h, PBMC 34days) are performed in a number of methodological and experimental variants v_i, globally collected in T, where:

$T = \{v \in T \mid v_j = \text{FunctVariants, EnrichMethods, } omics\text{Layers, ExperimentalBatch, j} = 1,..4\}$,

with:

$\text{FunctVariants} = \{t \in v_1 \mid t = \text{EMT, EMT.T2, whGO}, rw\text{GO}\}$, with EMT, EMT.T2, whGO, rwGO defined above;

$\text{EnrichMethods} = \{t \in v_2 \mid t = \text{DAVID, HPGM, GSEA}\}$, with DAVID enrichment analysis [19] only for GO categories; HPGM hypergeometric distribution [20] corrected for multiple hypothesis testing across gene setswith Benjamini Hochberg FDR [21], cutoff 0.05; GSEA [22], preranked mode, P value cutoff 0.05, q-value cutoff 0.05;

$Omics\ \text{Layers} = \{t \in v_3 \mid t = \text{mRNA, miRNA}\}$;

$\text{ExperimentalBatch} = \{t \in v_4 \mid t = 1,2\}$.

Each enrichment test t in each v in T has 3 possible outcomes x_i, $i=1,2,3$: $x_1=up$ for upregulated genes enrichment, $x_2=down$ for downregulated genes enrichment, $x_3=both$ for up and downregulated genes enrichment, these outputs are True if the enrichment is statistically significant and False viceversa. Depending on the experimental design, each spatiotemporal sample can be analyzed across different *t* in different *v* (not all samples are run in 2 batches or for two *omic* layers etc.).

Fig.1 E is the result of progressive summarization of the enrichment outcomes x_i. The first level of summarization is computed for each enrichment method t_i in v_2 with:

$$Eq.1 \begin{cases} up_{ti,v2} = (up \text{ XOR } down) \text{ AND } up \\ down_{ti,v2} = (up \text{ XOR } down) \text{ AND } down, i = 1,2,3 \\ both_{ti,v2} = (up \text{ AND } down) \text{ OR } both \end{cases}$$

Where $both_{ti,v2}$ is True also in cases where up and $down$ are True (within test –enrichment mehod- summarization). Namely, sample Z enriched for function Y showing up and $down$ significant enrichments computed with an enrichment analysis method is compatible with an overall *both* enrichment computed with the same enrichment method.

With a similar rationale, a second level of summarization is done for each variant $v_{j,}$ however, in in cases where up and $down$ are True the summary *both* enrichment must be False (across test summarization). Sample Z enriched up for function Y in batch 1 and enriched $down$ for the same function Y in batch2 must lead to no enrichment overall, as this would indicate that results in batch1 are not reproducible in batch2. This modifies Eq. 1 only for the last equation, and from this stage on leads to mutually exclusive x_i values (i.e. only one will be True):

$$Eq.2\begin{cases} up_{vj} = (up\,\text{XOR}\quad down)\,\text{AND}\,up \\ down_{vj} = (up\,\text{XOR}\quad down)\text{AND}\,down,\, j = 1,..,4 \\ both_{vj} = (up N\text{AND} down)\text{AND} both \end{cases}$$

The coherence score $C_x = X / |\{T\}|$, that multiplies the final logical score output in Eq.2 is used to visually grade the intensity of up (green) and $down$ (red) enrichments as shown in Fig. 1E of the main text:

Inflammation has a combinatorial biological meaning, i.e. physiologic when accompanying the early phase of healing and pathologic (chronic) in later stages (as well as being a hallmark of RA/CIA). To model this peculiar functional aspect, ongoing inflammation in Fig. 1E of the main text is depicted directly with the logical value of $up = |\{t \mid t == up, \forall t \in T\}|$ with a visually intuitive binary shade of red on top of other functions' grades.

Supplementary Figure

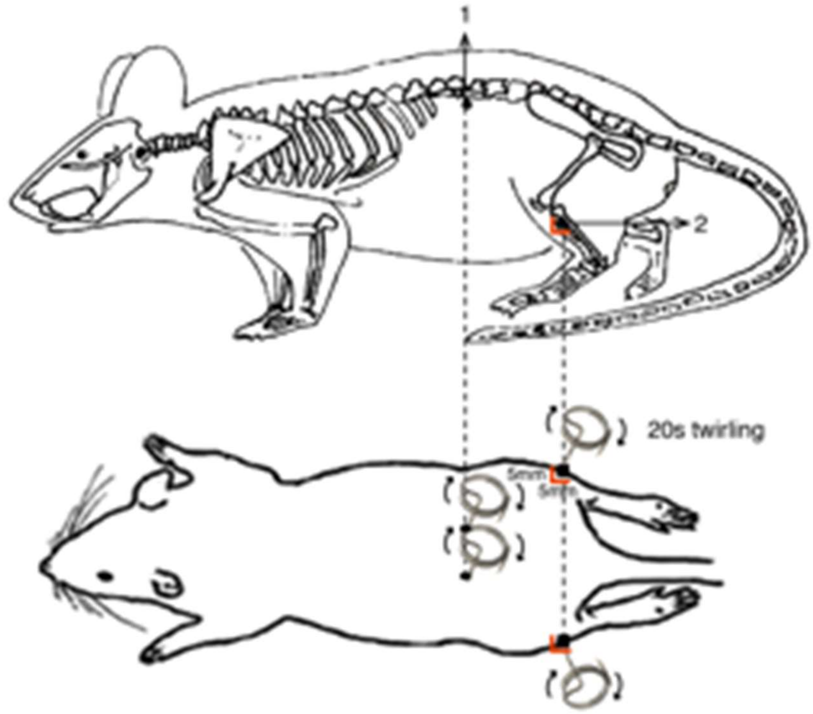

Figure S1. Schematic representation of the molecular stimulation (MS) applied. Four points identified through to anatomical landmarks (also referred to as "Shenshu" (BL 23) for point 1, and "Zusanli" (ST 36) for point 2) were selected, needled with ring-headed thumb-tack like stainless-steel needle (ϕ0.25mm*2mm, diameter*length, Hwato, Suzhou, P.R.C.) and stimulated for 20s with clockwise rotation every other day. Adapted from (He, T. F. et al. Electroacupuncture Inhibits Inflammation Reaction by Upregulating Vasoactive Intestinal Peptide in Rats with Adjuvant-Induced Arthritis. Evid-Based Compl Alt, doi:10.1155/2011/290489 (2011)) and (Xu, Y. D. et al. Proteomic analysis reveals the deregulation of inflammation-related proteins in acupuncture-treated rats with asthma onset.

Evid Based Complement Alternat Med 2012, 850512, doi:10.1155/2012/850512 (2012)) under Creative Commons License (https://creativecommons.org/licenses/by/2.0/legalcode).

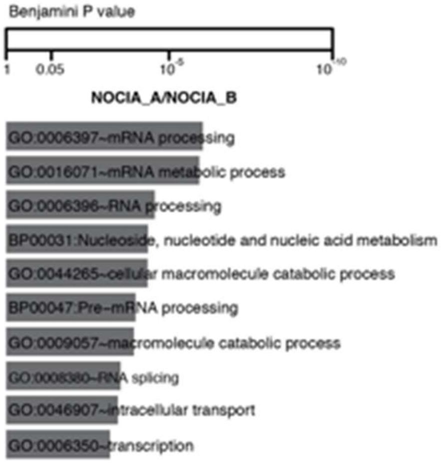

Figure S2 Functional enrichment NOCIA arm. Significant (top 10) enriched functional categories, using the same criteria adopted in Figure 1b. Coherently with the phenotypic aspects of the inflammatory stimulus (CIA) shown in Fig. 1a, no progression nor regression of inflammation and immune response are involved, nor are activation nor healing functions differential. Results refer to nucleic acids metabolism (transcription, splicing, processing, intracellular transportation) and biochemical transformations (catabolism) providing cellular components and energy for anabolic reactions, as part of the metabolic process for the physiologic self-maintenance of the cell.

Supplementary Table

Batch	Treatment A	Treatment B	Treatment effect Pr(>F)	Treatment : Time Pr(>F)
Batch1	ANE	MS	<0.01	<0.01
	ANE	MTX	<0.01	<0.01

	ANE	PLA	0.015	<0.01
	MS	MTX	0.627	<0.01
	MS	PLA	<0.01	<0.01
	MTX	PLA	<0.01	<0.01
Batch2	MS	MTXMS	0.061	<0.01

Table S1 ANOVA significance. Results of ANOVA to show significance of difference between treatments (for continuous data -Batch1 and Batch2) – response = CIA measurement. Results suggest that the only combination that has no significant difference between treatments (at the 5% level) is MS and MTX (batch 1) and 2 MS and MTXMS (batch 2). It is noted that there is a significant treatment to time interaction).

Sample	Therapy	Batch	Technology	Time points<br>***() only for batch1***
**Subcutaneous tissue**	AP, PLA, NOCIA	1	RNA-seq	0d, 1h, 34d
**Blood PBMC**	AP, PLA, ANE, MTX, NOCIA,	1,2	Microarray+ qRT-PCR	0d, (1h), 34d
**Feces**	AP, PLA, ANE, MTX, NORA	1	16S rRNA-seq	0d, 34d
**Blood serum**	AP, NOCIA	2	ELISA	0,18d
**Synovium**	AP, PLA, NOCIA	2	RNA-seq+ (qRT-PCR on markers)	0, 18d

Table S2 Sampling Summary. Summary of the samples, therapies, technologies and time points explored across two studies (batch1 and batch2) for the analysis of the molecular effects of MS on rats affected by CIA.

Supplementary Data

Data S1- Phenotypic data:

- Batch1 Arthritis categorical scores
- Batch1 Paws' thickness continuous measures
- Batch2 Arthritis categorical scores
- Batch2 Paws' thickness continuous measures

Data S2- PBMC mRNA differential and enrichment analysis:

- List of differential mRNAs (after to before therapy)
- Enrichment analysis with David therapy-wise
- Enrichment Analysis with David for MS-MTX
- qRT-PCR results

Data S3- Subcutaneous tissue mRNA differential and enrichment analysis:

- List of differential genes (after to before therapy)
- Enrichment analysis with David therapy-wise

Data S4- PBMC miRNA differential analysis and targets:

- List of differential miRNAs (after to before therapy)
- miRDB predicted targets of the differential miRNAs
- miRBase validated targets of the differential miRNAs
- miRNA&mRNA (includes predicted+validated differential miRNAs and differential mRNAs (from Supplementary Data S2)

Data S5- Subcutaneous tissue miRNA differential analysis and targets:

- List of differential miRNAs (after to before therapy)
- miRDB predicted targets of the differential miRNAs
- miRBase validated targets of the differential miRNAs
- miRNA&mRNA (includes predicted+validated differential miRNAs and differential mRNAs (from Supplementary Data 3)

Data S6- Spatiotemporal assays enrichment analysis:

- List of genes sets used for enrichment (reference sets)
- Enrichment analysis results

Data S7- 16S rRNA differential analysis, genera annotation, association tests:

- OTUs abundances
- Summary of differential fecal 16S rRNA analysis (Genus level) at 34 days (after to before)
- Differential genera annotated as eubiotic or dysbiotic.

- Statistical test association of the eubiotic/dysbiotic microbiome varied composition between treatments

Data S8- Synovial tissue mRNA differential and enrichment analysis:

- List of differential mRNAs
- Enrichment analysis with David
- qRT-PCR validation on 3 markers

1 Quast, C. *et al.* The SILVA ribosomal RNA gene database project: improved data processing and web-based tools. *Nucleic Acids Res* **41**, D590-596, doi:10.1093/nar/gks1219 (2013).

2 Rossello-Mora, R. & Amann, R. The species concept for prokaryotes. *FEMS microbiology reviews* **25**, 39-67, doi:Doi 10.1016/S0168-6445(00)00040-1 (2001).

3 Smyth, G. K. Linear models and empirical bayes methods for assessing differential expression in microarray experiments. *Stat Appl Genet Mol Biol* **3**, Article3, doi:10.2202/1544-6115.1027 (2004).

4 Lahti, L. *et al.* Associations between the human intestinal microbiota, Lactobacillus rhamnosus GG and serum lipids indicated by integrated analysis of high-throughput profiling data. *PeerJ* **1**, e32, doi:10.7717/peerj.32 (2013).

5 Bokulich, N. A. *et al.* Quality-filtering vastly improves diversity estimates from Illumina amplicon sequencing. *Nature methods* **10**, 57-U11, doi:Doi 10.1038/Nmeth.2276 (2013).

6 Robinson, M. D. & Oshlack, A. A scaling normalization method for differential expression analysis of RNA-seq data. *Genome biology* **11**, R25, doi:10.1186/gb-2010-11-3-r25 (2010).

7 Robinson, M. D., McCarthy, D. J. & Smyth, G. K. edgeR: a Bioconductor package for differential expression analysis of digital gene expression data. *Bioinformatics* **26**, 139-140, doi:DOI 10.1093/bioinformatics/btp616 (2010).

8 Abrahamsson, T. R. *et al.* Low diversity of the gut microbiota in infants with atopic eczema. *The Journal of allergy and clinical immunology* **129**, 434-440, 440 e431-432, doi:10.1016/j.jaci.2011.10.025 (2012).

9 Law, C. W., Chen, Y., Shi, W. & Smyth, G. K. Voom: precision weights unlock linear model analysis tools for RNA-seq read counts. *Genome biology* **15**, R29, doi:10.1186/gb-2014-15-2-r29 (2014).

10 Affymetrix. Statistical algorithms description document., (*2002*).

11 Lopez-Romero, P. Pre-processing and differential expression analysis of Agilent microRNA arrays using the AgiMicroRna Bioconductor library. *BMC Genomics* **12**, 64, doi:10.1186/1471-2164-12-64 (2011).

12 Simon, A. *FastQC A Quality Control tool for High Throughput Sequence Data*, 2010).

13 FASTX-Toolkit (http://cancan.cshl.edu/labmembers/gordon/fastx_toolkit/, 2009).

14 Marcel, M. Vol. 17 10-12 (EMBnet.journal, 2011).

15 Langmead, B. & Salzberg, S. L. Fast gapped-read alignment with Bowtie 2. *Nat Methods* **9**, 357-359, doi:10.1038/nmeth.1923 (2012).

16 Anders, S., Pyl, P. T. & Huber, W. HTSeq-a Python framework to work with high-throughput sequencing data. *Bioinformatics* **31**, 166-169, doi:10.1093/bioinformatics/btu638 (2015).

17 Friedlander, M. R. *et al.* Discovering microRNAs from deep sequencing data using miRDeep. *Nat Biotechnol* **26**, 407-415, doi:10.1038/nbt1394 (2008).
18 Griffiths-Jones, S., Saini, H. K., van Dongen, S. & Enright, A. J. miRBase: tools for microRNA genomics. *Nucleic Acids Res* **36**, D154-158, doi:10.1093/nar/gkm952 (2008).
19 Huang, d. W., Sherman, B. T. & Lempicki, R. A. Systematic and integrative analysis of large gene lists using DAVID bioinformatics resources. *Nat Protoc* **4**, 44-57, doi:10.1038/nprot.2008.211 (2009).
20 Rice, J. A. *Mathematical Statistics and Data Analysis*. (Stamford, Connecticut: Cengage Learning, 2007).
21 Benjamini, Y. & Hochberg, Y. Vol. 57 289–300 (J. R. Stat. Soc. Ser. B Methodol., 1995).
22 Subramanian, A. *et al.* in *Proc Natl Acad Sci U S A* Vol. 102 15545-15550 (2005).
